# Evolution of *Tat* Retrotransposons Reveals Mosaic Phylogenetic Patterns Among Land Plants

**DOI:** 10.3390/plants15152388

**Published:** 2026-08-04

**Authors:** Antonina Prokopeva, Kirill Plotnikov, Mikhail Biryukov

**Affiliations:** 1 The Federal Research Center Institute of Cytology and Genetics SB RAS, Lavrentiev Ave., 10, Novosibirsk 630090, Russia; 2 Institute of Natural and Social-Economic Sciences, Novosibirsk State Pedagogical University, Vilyuyskaya Str., 28, Novosibirsk 630126, Russia; 3 Department of Natural Sciences, Novosibirsk State University, Pirogova Str., 2, Novosibirsk 630090, Russia

**Keywords:** transposable elements, plant phylogeny, bryophytes, Gnetophyta, seed plant divergency, *Tat*

## Abstract

Transposable elements (TEs) are rarely used as phylogenetic markers because of their high copy number, frequent recombination, and potential for horizontal transfer. However, their long-term coexistence with host genomes suggests that some TE lineages may preserve information about the evolutionary history of their genomic environment. Here, we investigate whether *Tat* LTR retrotransposons of the Ty3/Gypsy superfamily retain a phylogenetic signal informative for deep plant evolution. We reconstructed the phylogeny of *Tat* reverse transcriptase domains among representative lineages of land plants, including bryophytes, lycophytes, ferns, gymnosperms, and basal angiosperms. The analysis revealed stable clusters characterized by both structural specificity, determined by the position of the additional ribonuclease H domain, and taxonomic specificity. In many cases, the *Tat* subclusters reproduced established host relationships at the genus and family levels, particularly within conifers, indicating a predominantly vertical mode of inheritance. The distribution of *Tat* lineages also preserved signals relevant to unresolved questions of plant phylogeny. Among seed plants, different *Tat* lineages reflected aspects of existing alternative hypotheses, including the association of gnetophytes both with conifers II and angiosperms. Interestingly, the strong separation between two major conifer groups, pines and cypress with yews, was observed. Among non-seed plants, the topology of *Tat* lineages highlighted the unique position of lycophytes by the preservation of multiple ancient transposon lineages associated with early diversification after aRNH domain acquisition. It also suggested that the origins of mosses and hornworts involved different patterns of lineage elimination from a common ancestor that carried all structures found in lycophytes. We propose a conceptual framework in which TE clusters are interpreted as collections of partially independent evolutionary lineages rather than as a single species tree. Under this view, *Tat* retrotransposons provide an additional layer of phylogenetic information that complements conventional molecular markers and reflects the mosaic nature of plant genome evolution. The present work, therefore, represents the first implementation of this approach rather than its final methodological form.

## 1. Introduction

**Figure 1:**
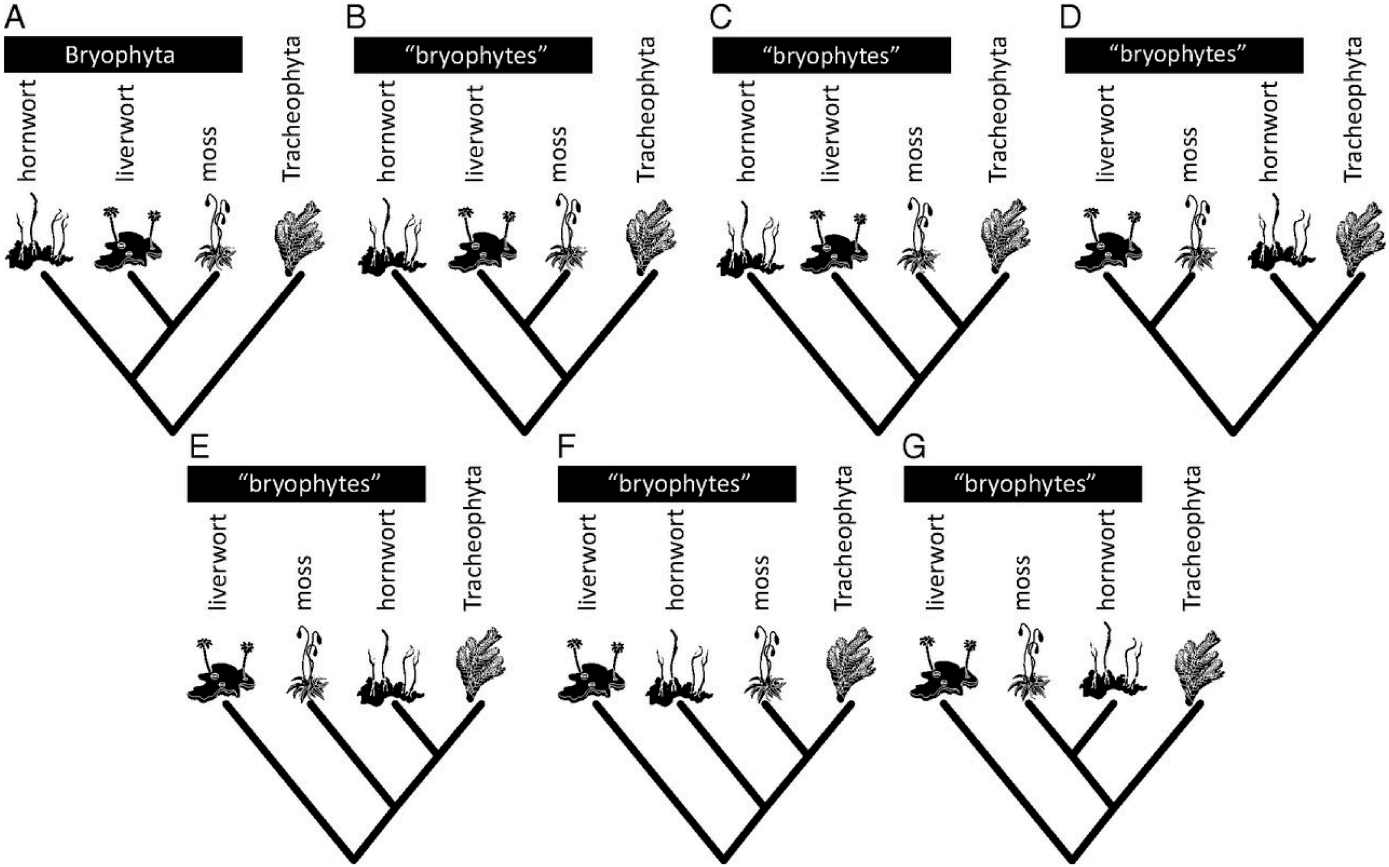
The scenarios of bryophytes’ evolution are taken from [3]. The seven alternative hypotheses were considered in the dating analyses. (**A**) Monophyletic bryophytes; (**B**) liverwort–moss sister clade to tracheophytes; (**C**) mosses, liverworts, and hornworts as successive sister lineages to tracheophytes; (**D**) a moss–liverwort sister clade to other embryophytes; (**E**) hornworts, mosses, and liverworts as successive sister lineages to tracheophytes; (**F**) mosses, hornworts, and liverworts as successive sister lineages to tracheophytes; and (**G**) a moss–hornwort sister clade to tracheophytes. Reproduced under the terms of the Creative Commons Attribution-NonCommercial-NoDerivatives License 4.0 International (CC BY-NC-ND).

**Figure 2:**
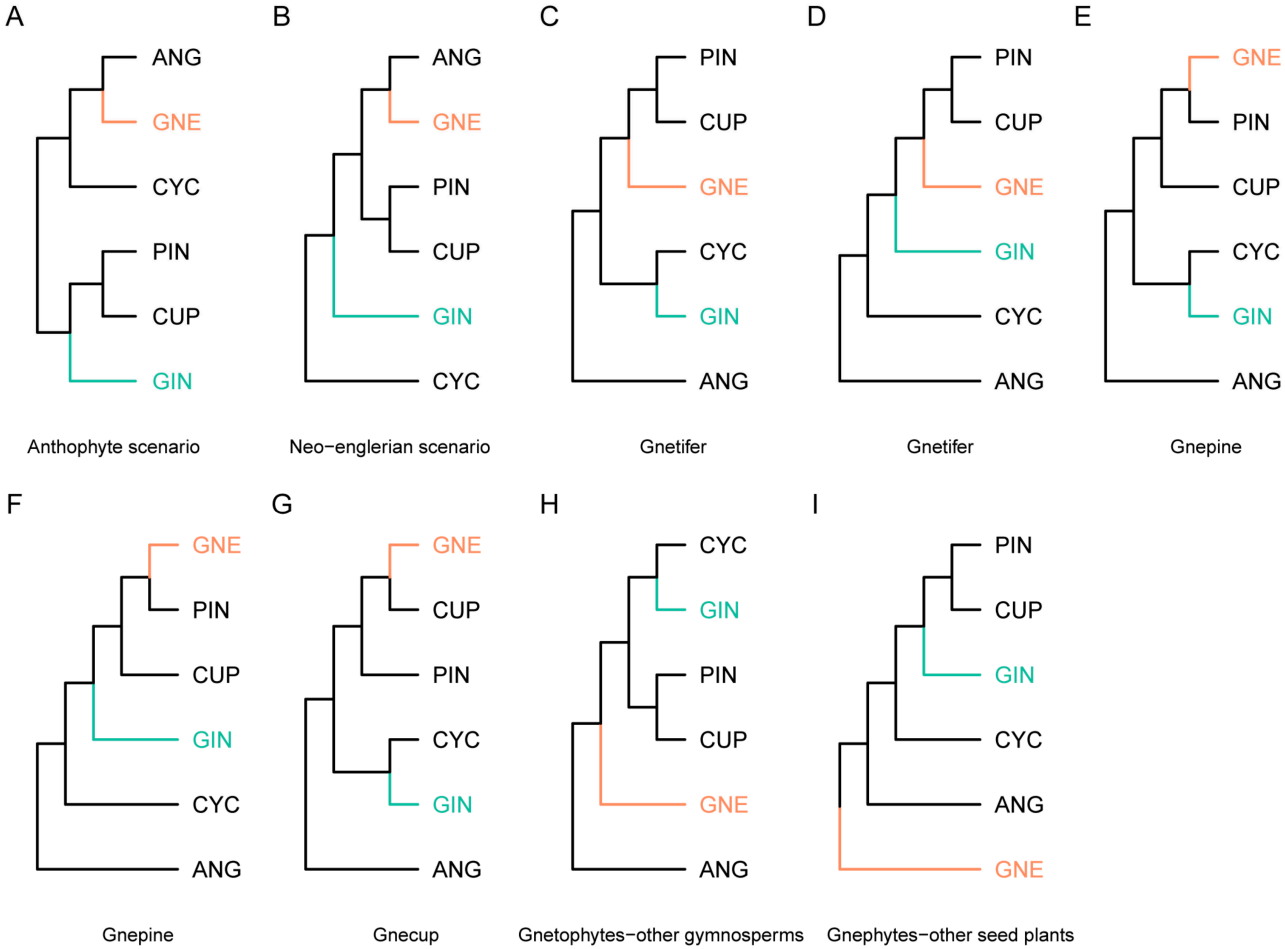
The scenarios of Gnetophyta evolution, taken from [13]. The different hypotheses on phylogenetic relationships of seed plant groups. (**A**) The anthophyte scenario, indicating that gnetophytes and angiosperms comprise a clade sister to Cycads; (**B**) the neo-englerian scenario, suggesting that gnetophytes and angiosperms comprise a clade sister to conifers; (**C**,**D**) the gnetifer hypothesis, implying that gnetophytes are sister to conifers, where (**C**) differs from (**D**) in the relationship to Ginkgo; (**E**,**F**) the gnepine hypothesis, suggesting that conifers are paraphyletic with gnetophytes sister to the Pinaceae, where (**E**) differs from (**F**) in the position of Ginkgo; (**G**) the gnecup hypothesis, indicating that conifers are paraphyletic with gnetophytes sister to cupressophytes; (**H**) the gnetophytes–other gymnosperms hypothesis, suggesting that gymnosperms constitute a monophyletic group and gnetophytes are sisters to a clade, including the rest gymnosperm groups; (**I**) the gnetophytes–other seed plants hypothesis, denoting that gymnosperms are paraphyletic and gnetophytes are sisters to a clade encompassing other seed plant groups. The colored branches and abbreviations display the jumping groups of the seed plants. Abbreviations: ANG: angiosperms; CYC: cycadophytes; GIN: Ginkgo; GNE: gnetophytes; PIN: Pinaceae; CUP: cupressophytes.

There remain some unresolved questions in plant evolution, such as both the early divergence of land plants and the radiation of seed plants. In particular, the order of divergence in the three major groups of bryophytes (Anthocerotophyta, Marchantiophyta, and Bryophyta), one of which is considered a Lycopodiophyta ancestor, remains a matter of debate [1,2,3,4,5]. Secondly, the position of Gnetophyta among seed plants has also been under discussion [6,7,8]. Morphological traits and classical molecular markers often provide controversial signals, preventing a final resolution of these issues. This inconsistency has clearly demonstrated that individual markers are not always capable of reflecting the complex picture of eukaryotic genome evolution. That is why there are plenty of hypotheses for these two unresolved problems. One or two groups of bryophytes (either Anthocerotophyta or Bryophyta) have been suggested to be sister to lycophytes in previous studies. In some cases, they have also been considered possible common ancestors of either two or all three groups of bryophytes, including a scenario where the lineage of all Tracheophyta taxa is sister to the last common ancestor of “bryophytes” (Figure 1) [3]. For gnetophytes (Gnetophyta), a presumably ancient group of gymnosperms presented by three divergent genus, no final taxonomical niche has been found: they have been suggested to be a sister group to one of two groups of conifers (Pinophyta divided into conifera I, consisting of Pinaceae, simply “pines”, and conifera II, consisting of Cuppresaceae and Taxaceae, simply “cypress”), to conifers together, as a sister group to angiosperms, or even as a basal seed plant taxa (all major scenarios are in Figure 2). To resolve these hypotheses, morphological comparison has previously been complemented by a few molecular markers. However, even approaches based on the analysis of a large number of marker and non-marker genes have also failed to provide a definitive resolution of these relationships [6,8,9,10,11]. Additionally, there have been some discussions about Cycadophyta and Ginkgophyta as separate [6,12] or sister taxa [9,10]. The main hypotheses are collected in Figure 1 and Figure 2.

Transposable elements (TEs) constitute a significant portion of plant genomes, far exceeding the volume of protein-coding sequences in a number of organisms [14,15,16,17,18,19,20,21]. Being one of the important sources of mutations, TEs introduce high genomic variability. On the other hand, they evolve closely together with the genetic loci they are located in, so they have the potential to carry information about the evolution of their hosts inside the TE-flanking genomic loci. Previously, a number of publications have reported that some TE groups contain highly conserved sequences among closely related groups of organisms carrying them or even behave in the same way as orthologous rRNA gene clusters, in which they sometimes take part in evolution and maintenance. With the seemingly vertical inheritance, their inter-TE phylogenetic distances are in correlation with the host’s ones [22,23,24,25]. TEs are used as markers in crop sciences to specify differences among varieties [26,27,28] and are suggested as indirect markers (TE-derived duplications, satellites, etc.) [24,29,30,31] but are rarely used as phylogenetic markers themselves. This is due to their ability for horizontal transfer (HT), high copying number, and involvement in recombination processes, which complicates the identification of orthologous sequences and may disrupt the signal of vertical inheritance [26,32,33,34,35]. So, the possibility of TE usage as common phylogenetic markers depends largely on the horizontal transfer possibility, which should be either excluded or mitigated.

In this study, we return to the question of whether TEs can be used in phylogenetic reconstructions. We propose that, despite the redundancy of copies and the possible contribution of HT, transposon clusters may retain a predominantly vertical signal. The crucial point is how to interpret them: not as a single tree of orthologs and paralogs, but as a collection of repeating clusters reflecting variations in the same evolutionary scenario. With this approach, TEs reconstruct not a single consensus phylogenetic tree of species, but instead provide an additional perspective on genome evolution, potentially capturing signals that depend on the genomic context in which the TEs are localized, and which evolves alongside them.

Thus, the clusters of TEs under consideration reflect not only their own evolution but also the evolution of the genomic regions they are located in.

To demonstrate this approach, we focused on TEs from the cluster *Tat* from Ty3/Gypsy LTR retrotransposons [36,37,38,39,40]. As the main difference from other Ty3/Gypsy, *Tat* is characterized by a heterogeneous lineage structure, distinguished by the position of the additional ribonuclease H (aRNH) domain in the transposon genome. The structure-specificity of lineages may indicate relatively limited recombination and, likely, a low frequency of horizontal transfer. Previously, it has been noted that, after their origin through HT acquisition from fungi [40], excepting the points of such recombination, *Tat* evolution resembles vertical inheritance [39]. Since the shifting of the plant *Tat* LTR retrotransposon starting point near a hypothetical common ancestor of terrestrial plants [39], all previous suggestions of the horizontal origin of different *Tat* lineages [37,38] could be revised to only one demonstrated horizontal transfer [40], with the following recombination and specification events as an alternative to HT interpretation. We reconstruct the phylogenetic structure of these elements in representative taxa and investigate how the patterns we observe relate to two unresolved questions in plant evolution:

(1) The origin of the major groups of seed plants—angiosperms, two groups of conifers, and ancient gymnosperms (Gnetophyta, Ginkgophyta, and Cycadophyta);
(2) The order of divergence of the three major groups of bryophytes: liverworts (Marchantiophyta), hornworts (Anthocerotophyta), and mosses (Bryophyta).

Rather than attempting to replace established phylogenetic markers, our goal is to assess whether TEs can provide an additional, independent layer of evidence and to explore whether their signal can help reconcile conflicting hypotheses by reflecting the composite nature of plant genome evolution.

## 2. Results

### 2.1. Discovered aRNH-Determined Structures

**Figure 3:**
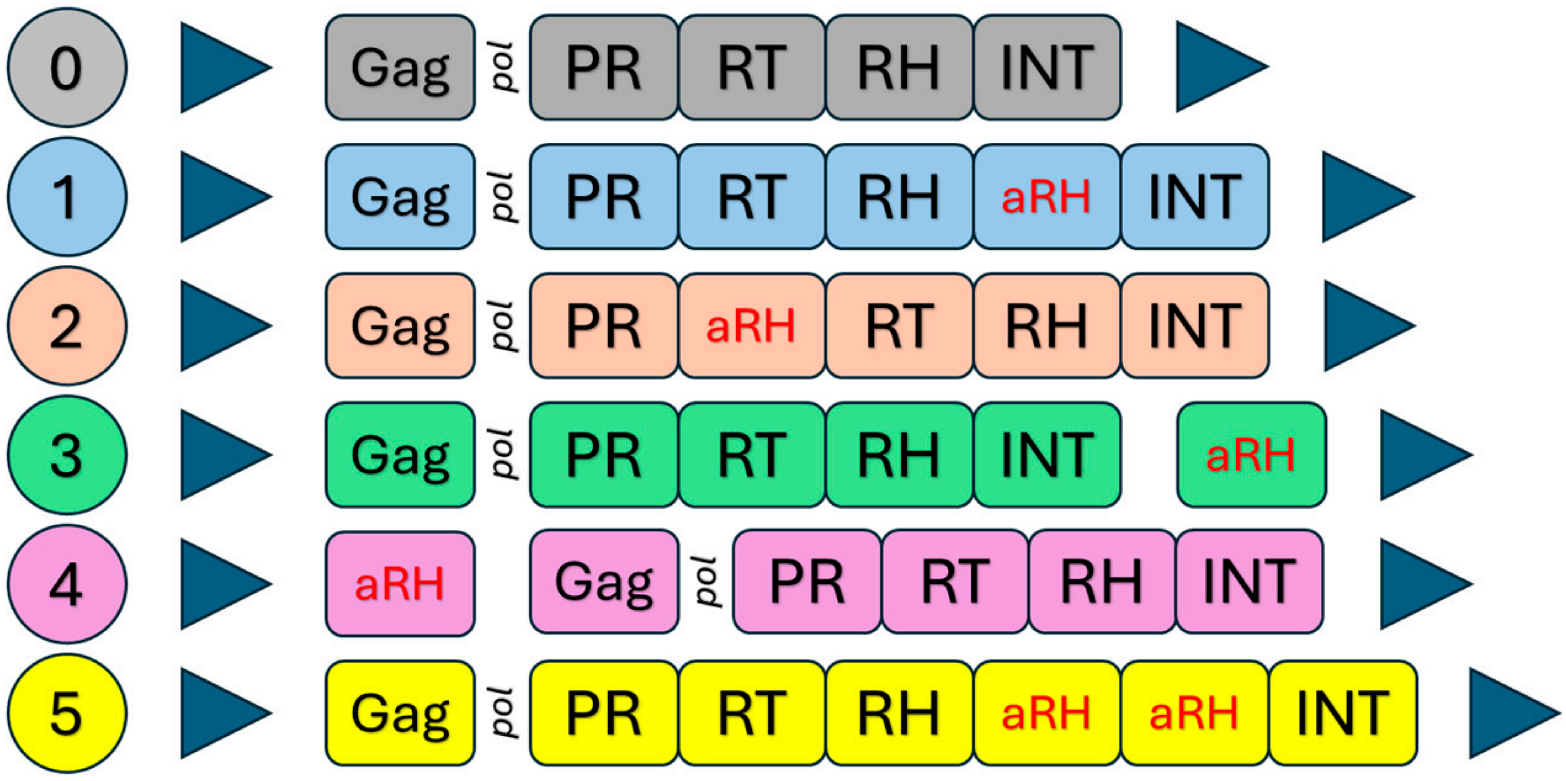
The structures of *Tat* LTR retrotransposons with different additional ribonuclease H domain positions. The domains are shortened as: PR—protease, RT—reverse transcriptase, RH—ribonuclease H, aRH—additional ribonuclease H, INT—integrase, and Gag includes all *gag* gene domains. All of the structures are presented by 2 or 3 open reading frames essential to their lifecycle activity: *gag*, *pol*, and solo *aRH*, which is pivotal for (3, post-integrase) and (4, pre-capsid) structures. The blue triangles are LTR (long terminal repeat) sequences. In the main text, the structures are called: 0—the “zero” structure (common for most Ty3/Gypsy representatives), 1—retroviral, 2—copia-like, 3—post-integrase, 4—pre-capsid, and 5—three-domain retroviral.

In our study, cluster *Tat* elements were detected in all examined groups of land plants. Previously, five structures with different positions and copy numbers in the aRNH domain, as well as a “zero” structure without the acquired domain, were identified within the *Tat* cluster [39]. The “zero” structure of *Tat* LTR retrotransposons fully corresponds to that of the common ancestor, its sister group *Athila*, before it acquired the *env* gene. We designated the non-“zero” structures (Figure 3) as follows: 1—“retroviral”, as it has been termed in previous studies [37,38], where the domain arrangement is similar to that of retroviruses, which also contain two ribonuclease H domains.

2—“copia-like”, where aRNH is located between the protease and the reverse transcriptase (like the integrase domain in Ty1/Copia elements, which is the main structural difference from its location downstream from revertase and native ribonuclease H in Ty3/Gypsy [41,42,43]).

3—“post-integrase”, aRNH is located downstream of the integrase domain of the TE genome. There were controversial reports about it being either part of the *pol* gene or as a separate open reading frame (ORF) in the 3′UTR [37,39].

4—“pre-capsid”, where the aRNH domain is located as an independent ORF in the 5′-UTR preceding all other domains (discovered by [39,40]).

5—three-domain retroviral, where three copies of the ribonuclease H domain (one native and two additional [39]) are in the “retroviral” configuration. In this study, it is considered a derivative of the classical retroviral structure and is not classified separately.

In most cases, the found clades demonstrated distinct specificity in both structure and taxonomic affiliation.

### 2.2. RT-Based Phylogenetic Tree of Tats

**Figure 4:**
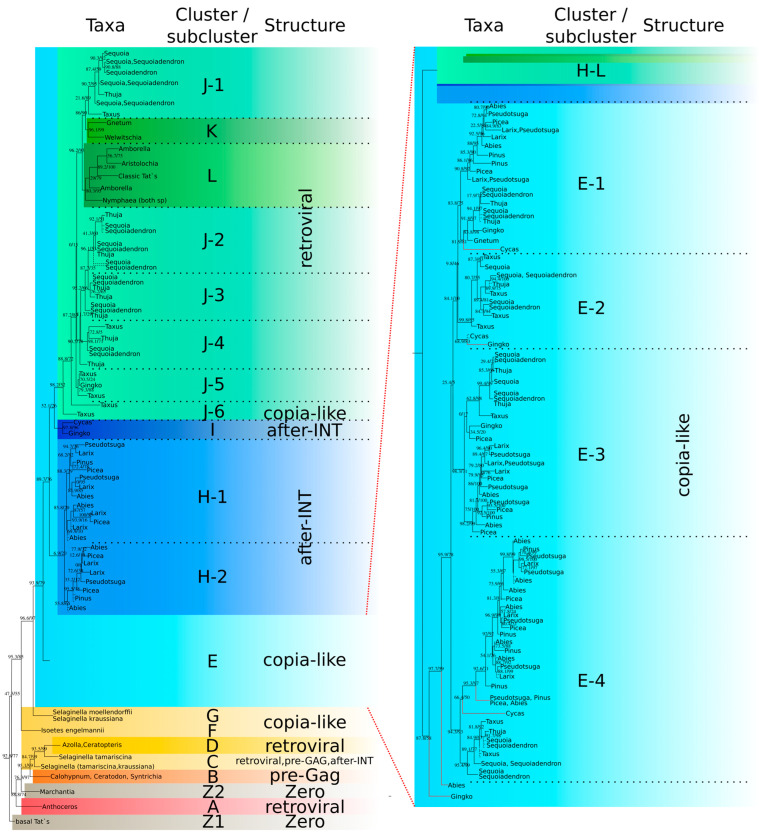
The RT-based phylogenetic reconstruction of the found *Tat* elements. The taxa are presented by genus names, except for lycophytes with additional species names (full taxonomy listed in Table S1). The red branches indicate lineages and parts of subclusters that are represented by a few lineages or lineages with partial RT sequence but have the potential to be considered not as artifacts but as lineages that once existed. The middle column indicates clusters within *Tat* identified in a previous study and their subclusters (for *Tat E*, *H*, and *J*) divided in the current study. The main difference from previous reconstructions is the location of *Tat F-G* clusters that previously were found between clusters *Tat E* and *Tat H-L*. The third column indicates one of the four main structures from Figure 3. The nodes are marked by aRLT/ufBoot support values.

We reconstructed the original phylogenetic tree (hereinafter we use the classification of *Tat* divided into clusters *Tat A-L*,*Z1-2* from a previous study [39]) with a reduced number of species, retaining all the major *Tat*-subdivided clusters from the previous study (Figure 4). The analysis included one to five representatives of three groups of “bryophytes” (Marchantiophyta, Anthocerotophyta, and Bryophyta), lycophytes (Lycopodiophyta), ferns (Polypodiophyta), Ginkgos (Ginkgophyta), Cycads (Cycadophyta), gnetophytes (Gnetophyta), pines (Pinophyta, conifera I), cypresses and yews (Pinophyta, conifera II), and basal angiosperms (Magnoliophyta). Hereinafter, we use a classification of Pinophyta divided into two groups: “conifers I” (pine families) and “conifers II” (cypresses and yews), which corresponds to the observed differences in the structure of the clades they form [9,12]. A larger number of modern seed plant species was selected to assess the verticality of the trait of inheritance within the clades they form (by comparing their branching patterns with the established phylogenetic framework for these genera), as well as for lycophytes, which exhibit deviations from classical plant phylogeny. The resulting tree is depicted in Figure 4 (and the complete structure is shown in Figure S1). At the structural level, we also observed *Tat* clusters, corresponding to those previously identified [39]. Globally, *Tat* retained its division into three major branches:

(1) The *Tat Z1* (“zero”) branch, represented by elements lacking aRNH, previously reported as basal *Tat* lineage;
(2) The non-seed plant branch, comprising the *Tat A–D* and *Z2* clusters;
(3) The *Tat E–L* branch, represented by clusters of seed plants and lycophytes.

The clusters were further divided into subclusters in cases of either single taxon specificity or as possible “subtrees” (defined further in Section 3.1). The numeration of subclusters is shown in Figure 4.

### 2.3. Tat Branch of Predominantly Seed Plants

The lycophytes and seed plants are divided into two basal lycophyte clusters (*Tat F* and *G*) and combined seed plant clusters (*E* and *H–L*). The first clade to diverge after the lycophytes is a large cluster with a copia-like structure (*Tat E*, Figure 4, light blue background). It is represented by all gymnosperm taxa, including ancient groups. The second half of the large seed plant branch includes retroviral and post-integrase structures, as well as a single-taxa copia-like structure that has been preserved (or has just emerged) in a single species, located at the boundary between them. Here, the basal branches first diverge into the conifer I cluster with a post-integrase structure (*Tat H*), followed by the cluster of ancient gymnosperms (*Ginkgo* and *Cycas*) with a similar structure (*Tat I*). Both are marked with a dark blue background in Figure 4. Unlike the ancient gymnosperms, conifers I are no longer found in this clade of seed plants, occupying the outermost positions within it. Next comes a single subcluster separating elements with different structures, consisting solely of *Taxus* copia-like elements (part of the paraphyletic cluster *Tat J*). Then, there are only retroviral structures. Following the branches for *Taxus*, *Ginkgo*, and conifers II, three adjacent clades are formed: angiosperms (*Tat L*, yellow green in Figure 4), *Gnetophyta* (*Tat K*, brown green in Figure 4), and conifers II (the remaining part of *Tat J*, *J-1*). The identified clades are represented on a tree, reflecting the relationships among the seed plant taxa (Figure 4, blue background, and the others nested within it).

Throughout the branching process, several levels of subclusters can be identified in virtually all the clades mentioned. Species-specific TE clades tend to form primarily in the neighborhood of clades from closely related taxa. Mixed clades (consisting of elements from more than one neighboring species, carrying them without strong separation) are periodically adjacent to or situated between such pairs of clades. As the analysis progresses from lateral species-specific clades to their combinations, subclusters form, which may then recur multiple times in the reconstruction.

The pattern is observed in clusters *E* and *H* for conifer I, where *Pseudotsuga* elements are consistently grouped closely with *Larix* elements and *Picea* elements with *Pinus* elements, while *Abies* is typically located at a distance from the others within individual subclades. This pattern corresponds to the current understanding of the phylogenetic relationships among the genus of conifers I.

A similar pattern is observed in a group of conifers II. Here, of the four selected species, three belong to the Cupressaceae family and one species represents a different family—the Taxaceae. Elements of two closely related genera, *Sequoia* and *Sequoiadendron*, are grouped together. Then, elements of the *Thuja*, a more distant relative of the *Sequoia* among the cypresses, join them basally, while the yew (*Taxus*) occupies a further, more distant position.

The diversity in angiosperm elements is represented in only one clade on the tree (corresponding to cluster *Tat L*). This cluster is composed primarily of classical *Tat* elements (*Tat4-1*, *Ogre*, *Grande1-4*, and others). In this case, four genomes were selected from representatives of three different ancient groups [44] of angiosperms: the Amborellaceae (*Amborella trichopoda*), Nymphaeaceae (*Nymphaea colorata* and *Nymphaea thermarum*), and Aristolochiaceae (*Aristolochia fimbriata*) families. Together, these taxa form two subclusters, each of which includes two smaller family-specific subclusters. Each of the two subclusters contained one taxon-specific cluster of Amborellales and one of the other taxon (elements of two Nymphaeaceae species were mixed).

### 2.4. Tat Elements of Non-Seed Plants Are Distributed Between Two Branches

Two lycophyte clusters (*Tat F* and *G*) are sister to the seed plant clade. The second *Tat* branch contains representatives of all other non-seed terrestrial plants, including another paraphyletic lycophyte cluster (*Tat C*). Here, apart from lycophytes, each taxon is represented by a separate branch with a unique structure. In this context, we interpret the case of Anthocerotophyta (*Tat A*) with retroviral structure and its derivative three-domain retroviral structure as simple TEs with a retroviral structure. From the liverworts (*Tat Z2*)—which, due to their topology and lack of aRNH, can be considered as being close to the state preceding the acquisition of aRNH by *Tat* elements—different clusters branch off in two directions. One branch directed to the seed plant elements along this path shows that hornworts (*Tat A*) first branch off, followed by two groups of lycophytes (*Tat F* and *G*, basal to the seed plants). In the other direction, mosses (*Tat* B), ferns (*Tat* D), and located between them, the paraphyletic cluster of lycophytes (*Tat C*), represented by both post-integrase (mosses) and retroviral (ferns) structures, diverge.

### 2.5. Tat Z1

The third, most basal *Tat* branch, which is not yet grouped with the sisters to all *Tat* clusters, *Athila*, but does not contain an additional domain, has been designated “zero”. According to previous work [39], it contains elements from most taxa that contain other *Tat* elements, although these elements have been fully lost in some of them. This branch may correspond to the *Tat* cluster prior to the acquisition of the aRNH domain and, thus, represents the “root” from which the *Tat* TEs of non-seed plants originated. Due to the nonspecific loss of elements and the inability to identify structure-specific subclusters among them, it was not included in further analysis. It is located basally on the tree and shows the greatest similarity to elements from the Marchantiophyta, since they are also characterized by the absence of the aRNH domain (cluster *Tat Zero 2* from the previous study). In Figure 4, this is represented by the basal gray branch.

## 3. Discussion

### 3.1. Conceptualization

In most of the subclusters discussed in this study, a distinct specificity is observed at different levels: the formation of species-specific small clades (for example, *Sequoia*, *Sequoiadendron*, *Thuja*, and *Taxus* clades in *J-1*), the presence of higher taxa-specific clusters in the main plant groups (clusters *Tat A*, *B*, *C*, *D*, *K*, *L*, and *Z2*) and structure-specificity with no admixture by another structure inside the major part of subclusters and clades. In many cases, additional groups of elements are distinguished within large clusters, separated by the composition of the taxa they contain and the nature of their distribution on the tree, which allows such subclusters to be considered as independent phylogenetic units, reflecting the relationships between a limited number of taxa. At the level of taxonomic groups with established systematics (primarily at the genus level), most of these subclusters are consistent with the known phylogeny of their hosts.

The main exceptions to this pattern are small groups of elements and isolated branches, which are difficult to interpret. These include, in particular, elements that form long branches (in most cases the RT domain is less than 50% of its normal length) and are represented by a few copies (typically no more than ~10 sequences). Such cases often correspond to degraded or fragmented elements and can be considered as reconstruction artifacts (shown in red in Figure S1; examples are single lineages of *Welwitschia* in *J-1* and of *Taxus* in one of the *Amborella* clades in *Tat L*).

Therefore, an additional criterion for interpreting transposon clusters can be proposed. Active elements are typically represented by a set of closely related copies that form the “core” of the cluster/clade, whereas older and partially degraded copies form its periphery (“shell”). Clades lacking such a “core” and “shell” combination are more likely to reflect either artifacts or residual traces of previously existing lineages. The idea of “core-and-shell” of clusters will be discussed below and depicted in Figure S2.

In rare cases, such isolated elements could theoretically result from horizontal transmission. It should involve TE copies from distantly related species located together, avoiding the species that, in the case of vertical evolution, are expected to be between them. If such an event has happened, the newly transmitted element should undergo extensive amplification to reach the abundance needed to be fixed, further leading to the formation of a consistent “core” and “shell”. Also, there should be good node support, uniting two species and indicating the derivatization of one species from another. If elements are whole (non-degraded) and even have differences in domain structure that can’t be found in the closest leaves, this could be an additional indicator of HT capability. However, they often have damaged domains, lack an inherent “core”, and have low frequency, which makes them unlikely to fixate and suggests that they would have a significant impact on the reconstructed tree topology, allowing researchers to exclude them from further analysis without a significant loss of information.

The clades with few elements (*Cycas* lineages in *E-1* and *E-4*, and *Ginkgo* lineage in *E-2*) play an intermediate position between artifact lines and lines that can be interpreted as fossils from previously existing common ancestors but then eliminated in most taxa. In most cases, the absence of distinct “core” and “shell” structures serves as grounds for classifying them as artifacts. If such clades are located between large species-specific clade agglomerations, they can be regarded as “noise” that carries no interpretable information. In some cases, we call those clades “separators” (*Pinus* lineages in *E-1*), which indicate the possibility of treating neighboring groups as independent “subtrees”. Sometimes they are problematic for their interpretation because they can be used as a part of both subtrees that they are connected to. A situation arises when such clades are embedded within or between small subclusters: in this case, they may be part of the local topology and should be considered in its interpretation. Such cases were encountered in our study; however, the instability of their positions when reconstruction parameters are varied indicates the limited reliability of the corresponding topologies.

To interpret the resulting clades, let us consider a general conceptual model linking the divergence of transposable elements to speciation processes. Within the framework of a simplified scenario involving predominantly vertical inheritance, the following patterns can be expected:

(1) An active transposable element that is not in a dormant state replicates itself, forming a set of closely related copies. In the early stages, these copies are highly conservative and form the “core” of the clade, consisting of active or recently emerged elements.
(2) Over time, some copies diverge and undergo degradation as a result of stochastic mutations and defensive mechanisms by the host genome. This leads to the formation of the clade’s “shell”—a collection of older and diverged elements that have often lost their autonomy.
(3) Individual elements from this periphery may give rise to new transposon lineages, which, under favorable conditions, form their own differently evolving clusters. However, most such lineages do not become established and are eventually eliminated, leaving only fragmentary traces (“fossils”) in the genomes, which we detect as artifacts.
(4) During speciation, each of the lineages of transposable elements present in the common ancestor is inherited by the daughter species. Subsequently, these lineages may diverge independently, leading to the formation of species-specific clades descended from a single ancestral lineage.
(5) Consequently, in a case of predominantly vertical inheritance, phylogenetic reconstruction is expected to reveal both species-specific clades and clades comprising elements from several related taxa, reflecting their common origin and heterogeneous rates of divergence.

Thus, the set of clades identified for a group of related species can, in some cases, be viewed as a hierarchical system of nested “subtrees” corresponding to different lineages of transposable elements. We will use this model to interpret the observed topologies.

### 3.2. Species-Specific Tendency at Genera Level Represents Host’s Relations

If mixed clades (for example, clades of *Sequoia* and *Sequoiadendron*, with optional *Thuja*, in subclusters *Tat J-1-4*) are excluded from consideration, for both groups of conifers, the taxonomy of their hosts is generally reproduced at the genus level. However, the mixed clades themselves should not necessarily be viewed as a deviation from this pattern.

**Figure 5:**
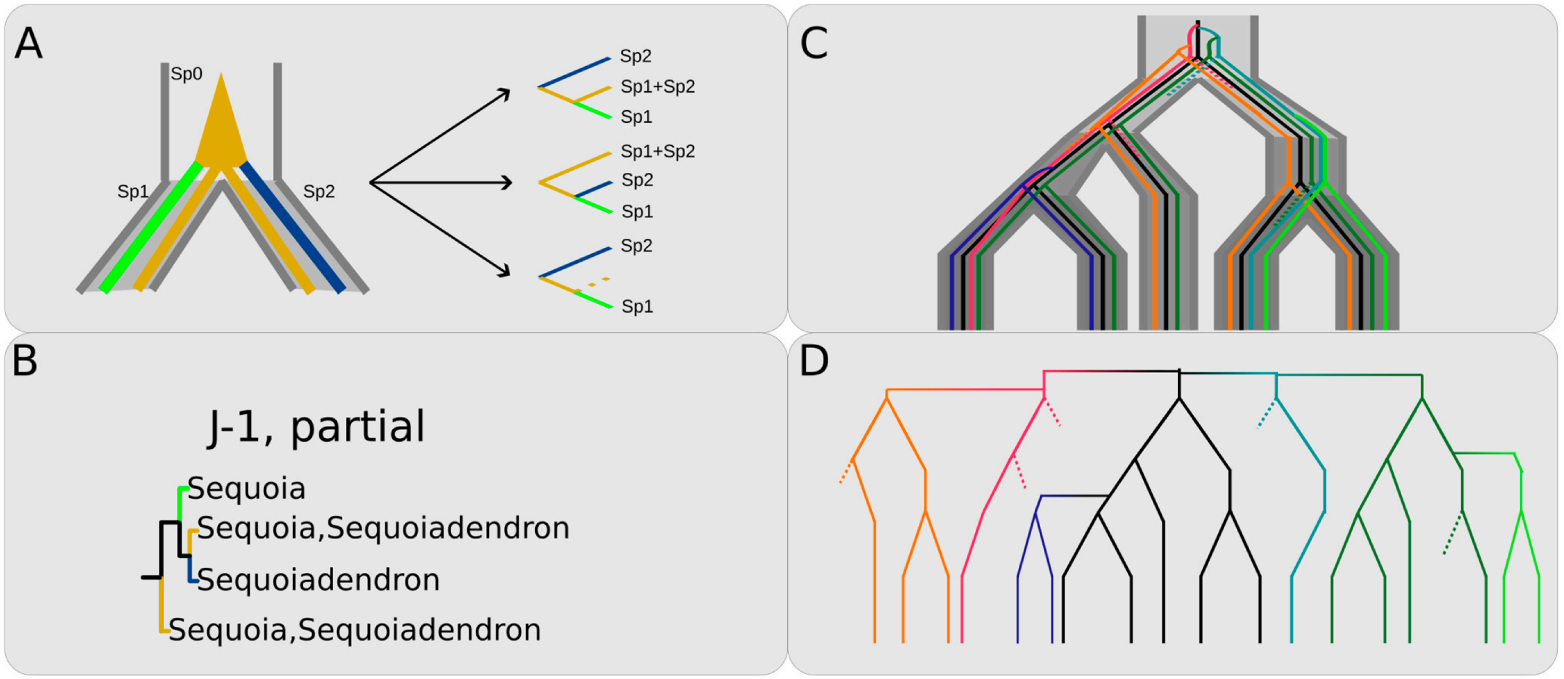
The main conceptual ideas of TE inheritance via specification. (**A**) The fate of the descendant TE lineages after the parental species (Sp0) is divided into two daughter ones (Sp1 and Sp2). The lineages that evolved more neutrally and closer to the element’s “core” (ocher) can either form a mixed clade of TEs (from both Sp1 and Sp2 genomes) with different positions on the tree or become eliminated. Elements from the “shell”, being more divergent from the “core”, are able to form new species-specific clades. (**B**) An example of two scenarios with the species-specific and mixed clades from (**A**). Here, two mixed clades are formed, both between and basal to species-specific ones. (**C**,**D**) The complex evolution of clades of elements via several rounds of specification from one lineage founder to different lineages that form clades reproducing the host’s phylogeny fully or partially. (**C**) The scheme of the TE phylogeny nested into the host phylogeny tree in the case of no HT evidence. One parental species caught the founder element (black lineage) and then evolved into 5 new species. (**D**) The unwrapped scheme of the TE lineage divergence from (**C**). The black TE lineage was saved in all daughter species and produced lateral lineages (different colors), which represent host-specific elimination events. Some cases, like the pink and light blue blocks, reflect single-genome remnants of lineages that failed to be inherited by all descendants of the host–founder species. They can be used as separators for subtree obtaining. Other lineages, like the light green and dark blue ones that originate later, could be both complementary for sister lineages (green and light green) and as species-mixed lineages. Dashed lines are evolutionary points of element extinction.

On the contrary, mixed clades located adjacent to species-specific ones may reflect TE lineages that existed in the common ancestor of the corresponding species and have been retained in several descendant taxa. In this case, the co-occurrence of species-specific and mixed clades appears to be a natural result of the long-term vertical evolution of *Tat* elements (Figure 5A).

Thus, the presence of an intermediate or basally located mixed clade between two species-specific clades may reflect one of the expected scenarios for TE divergence in closely related species (Figure 5B; using conifer II elements from the *Tat J-1* subcluster as an example). Such a distribution does not contradict the phylogeny of the hosts; on the contrary, it may further indicate the phylogenetic proximity of the corresponding element lineages.

Scenarios in which mixed clades are absent allow for a more significant number of interpretations. They may reflect either the independent divergence of the TE lineages following the divergence of the host species or the multiple elimination of ancestral lineages in some of the descendants.

Such dynamics are consistent with the notion of a long-term “arms race” between transposable elements and host genomes. In this context, isolated, species-specific clades, not accompanied by closely related lineages in related species, can be viewed as examples of the successful preservation and further divergence of individual TE lineages within specific genomes.

In our reconstruction (Figure 4), such isolated species-specific clades are most clearly represented by elements from *Abies* (conifers I), *Taxus* (conifers II), and *Ginkgo*, particularly in the basal regions of the *Tat E* and *Tat J* clusters. A schematic interpretation of such scenarios of TE–host coevolution is presented in Figure 5C,D. Among them, the *Abies* and *Ginkgo* elements at the base of *Tat E-4* can be considered examples of lineages preserved from the radical scenario in the blue block (preservation in only one representative). The multiple *Taxus* lineages at the base of *Tat J* (*J-5,6*) can be considered a reflection of the repeated occurrence of this scenario at different stages of lineage divergence within *Tat J.* The combined subcluster of *Ginkgo* and *Taxus* elements (*J-5*) corresponds to the scenario shown by the red block in Figure 5C,D. In light of this interpretation, some lineages previously considered to be at the borderline of artifact distance can be regarded as surviving evidence of more ancient states.

### 3.3. Conifers II Group Reproduces Host’s Phylogeny

**Figure 6:**
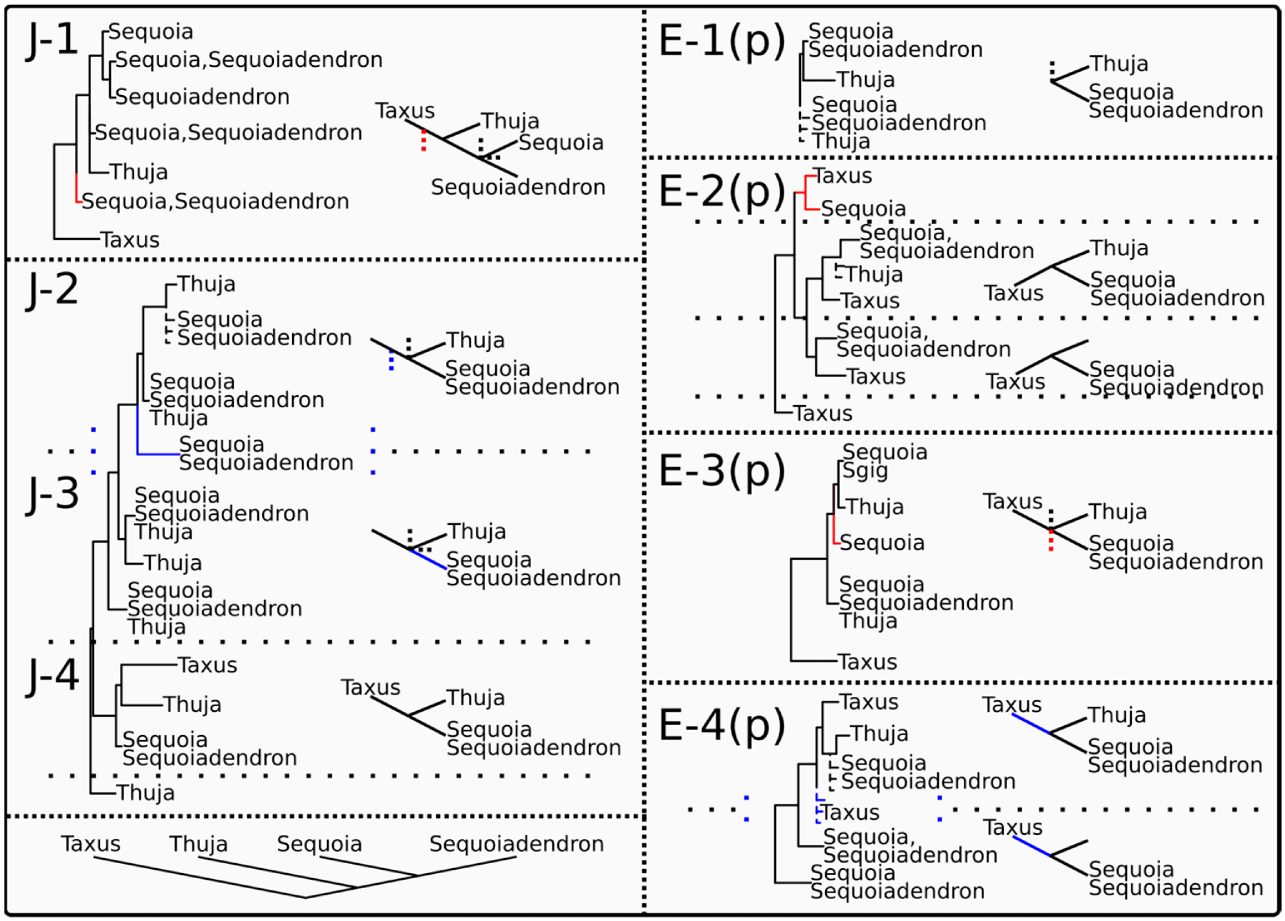
The main conifer II subclusters from the full *Tat* phylogeny (from Figure 4) and their unrooted simplified subtrees. The scheme presents the following subclusters: *J-1*, *J-2*, *J-3*, and *J-4*, and parts of *E-1*, *E-2*, *E-3*, and *E-4*. Mixed clades are presented as three-dotted lines from the nodes between the relative species-specific clades. The blue lines are lineages that could have dual membership in unrooted trees. The red lines are highlighted in the case of clades interrupting the simplest interpretation, meaning that there should be another split into a subtree (dotted at the subtree). Additionally, the scheme of phylogenetic relationships for the 4 studied species of conifers II (according to the literature [6,45]) is presented in the bottom left block. There are only 3 cases of unexpected clades (red) in *J-1* (could mean an additional TE lineage for Cupressaceae without Taxaceae), *E-2* (an additional subtree of two species is uninformative), and *E-3* (an unexpected *Sequoia* clade, and the *Sequoiadendron* one supposed to be eliminated). In all other cases, unrooted simplification matches the host’s phylogeny.

Let us examine the conifers II subclusters more thoroughly (Figure 6). In the case of *Sequoia* and *Sequoiadendron* TEs, we observe both the formation of distinct species-specific clades and their partial overlap, reflecting the coexistence of independent lineages and lineages inherited from a common ancestor that have not undergone significant divergence.

Adjacent to them are elements from *Thuja*—a more distantly related member of the Cupressaceae. In most cases, TEs form their own basal clades. Isolated instances of the admixture of *Thuja* elements with ones from *Sequoia* and *Sequoiadendron* are systematically present, but they do not disrupt the expected phylogenetic relationships among species. In particular, there are no instances in which elements of one species are closer to elements of a more distantly related taxon than to elements of their closest relatives. Such a pattern would be expected in the case of horizontal transfer. Instead, there are only cases with no elements from closer taxa, meaning that they are more likely to be eliminated, and single small-numbered lineages without a “core” and “shell”, which are counted as artifacts. The observed distribution, however, is consistent with the preservation of lineages inherited from a common ancestor and their subsequent divergence within species. This makes it unlikely that these events can be interpreted as the result of horizontal transfer followed by fixation and allows them to be considered either as artifacts or as unstable elements of the incomplete topology.

A similar pattern is observed for the yew (*Taxus*)—a member of a different conifer II family (Taxaceae). Its elements form their own clades, which are basal relative to the Cupressaceae group, consistent with the phylogenetic distance that should be expected. Cases of mixing between elements of different families are also observed; however, after excluding artifact lineages, such elements do not form stable groups. This indicates the absence of a reproducible signal that could be interpreted as the result of horizontal transfer and allows us to consider them as artifacts or a consequence of topological instability.

In total, we selected 10 subtrees of conifers II from eight *Tat* subclusters and their parts. The incongruity between subcluster and subtree number (*E-2* and *E-4*) is presented as an example of nested subtrees and an unfinished separation into subtrees. Five of them were fully representative of the expected host phylogeny (in one case even with *Sequoia* and *Sequoiadendron* species-specific clades, whereas in the other four cases, there were only mixed clades of these two species). In three cases, there were no *Taxus* representatives and no *Thuja* in two other subtrees. In each case, the second species of that pair was located separately from the *Sequoia* and *Sequoiadendron* pair, eventually counted as also representing host phylogeny. Three exceptional cases (highlighted in red in Figure 6) were interpreted as either additional lineages in partially inappropriate positions (in the case of *J-1* and *E-3*), or a possibly existing lineage suffering from elimination that failed to evolve into separate subtrees because of the loss of two or three taxa (case *E-2*). From these exceptions, only the case of *E-2* may be counted as another subtree, leading to a binomial test of 10 of 11 (*p*-value = 0.00013) or 8 of 11, if not considering trees of the other two exceptions (*p*-value = 0.00882).

Thus, except for a single *Taxus* subcluster (*J-6*) with a copia-like structure, located in *Tat J* (and adjusted retroviral structure subclusters) at the boundary between the *Tat H-I* (after-INT structure) and *J-K-L* (retroviral structure) clusters, the distribution of TEs in conifers II generally reflects vertical evolution and is consistent with the systematics of their hosts.

### 3.4. Conifers I Group Also Resembles Hosts’ Phylogeny

**Figure 7:**
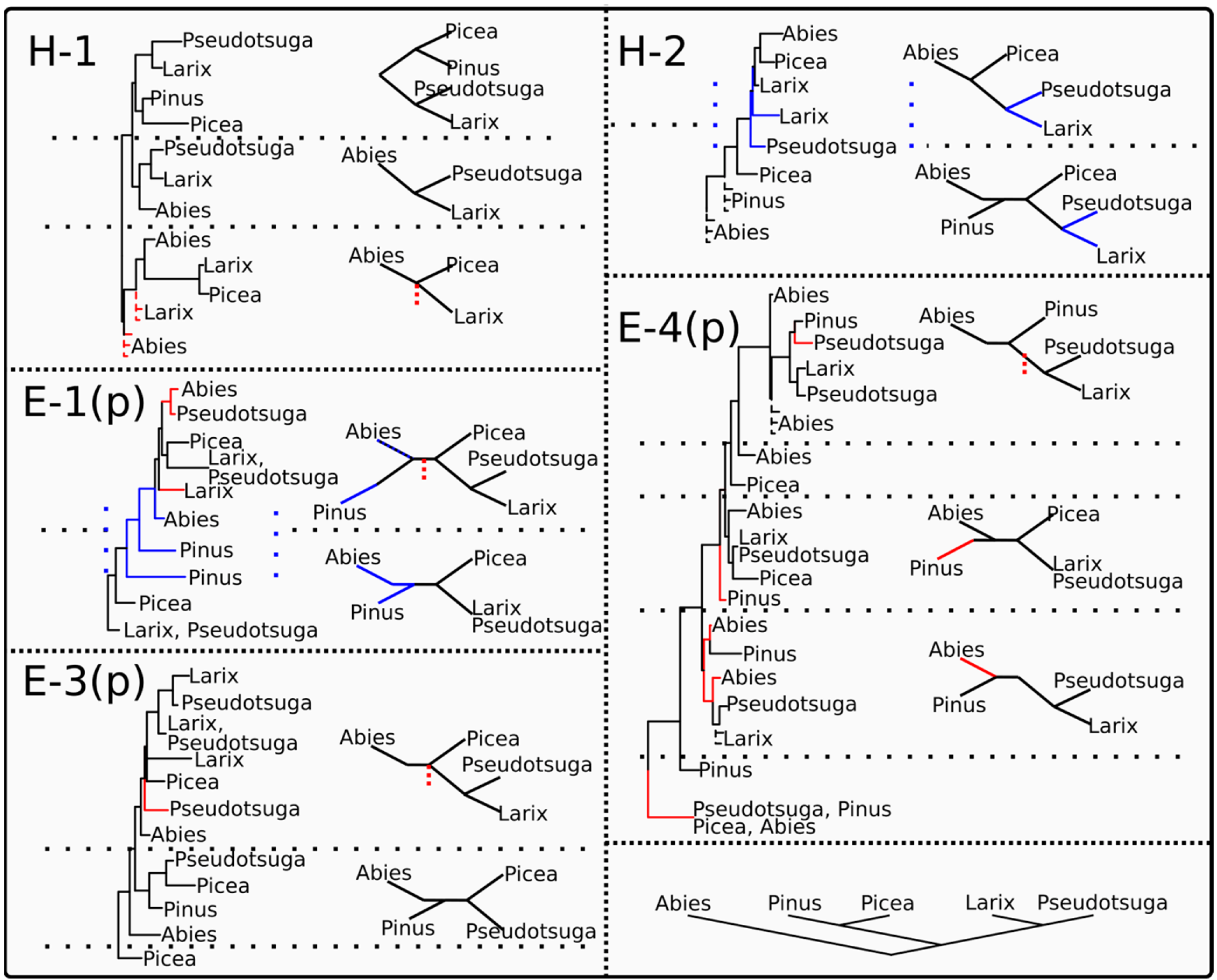
The main conifera I subclusters from the full *Tat* phylogeny (Figure 4) and their unrooted simplified subtrees. The scheme presents the following subclusters: *H-1* and *H-2*, and parts of *E-1*, *E-3*, and *E-4*. All of the mixed clades are counted as part of the relative species-specific clades, according to this paper’s concept. The blue lines are lineages that could have dual membership in unrooted trees. The red lines are highlighted in case of clades interrupting the simplest interpretation, meaning that there should be another split into a subtree (dotted at the subtree). Additionally, the scheme of phylogenetic relationships for the 5 studied species of conifers I (according to the literature [6,46]) is presented in the last block. Subcluster *H-1* is split into 3 subtrees, all in agreement with the host’s phylogeny. In *H-2*, the clades of *Larix* and *Pseudotsuga* are considered as parts of both subtrees, where they are extremely remote from *Abies*, while *Picea* and *Pinus* are between. In part of *E-1*, the block of *Abies* + *Pseudotsuga* is supposed to be another subtree (but uninformative because of only two species); elements from *Abies* and *Pinus* are parts of two other subtrees, where they are located remotely from other species. In the *E-3* partial, there are two subtrees correlated to the host’s phylogeny, except for interruption by the *Pseudotsuga* clade, which is supposed to be closer to the *Larix* one. In the *E-4* partial, one *Pseudotsuga* cluster in the first subtree can be counted as interruptive, but because of one additional clade of the same species nearby, it may be considered as a duplicated lineage evolved closer to *Pinus* (so the subtree has no changes). The two other subtrees place *Pinus* and *Abies* at a distance from other species.

The situation is more complex with the conifers I group (Figure 7). On the one hand, in most cases, the elements also form predominantly species-specific clades and less species-mixed clades. At the same time, there is a strong correlation with the phylogeny of the hosts: TEs of the genus *Larix* are located closer to the *Pseudotsuga* ones, while the elements of *Pinus* are sometimes closer to *Picea* and sometimes to *Abies*, which most often occupies the position furthest from *Larix* and *Pseudotsuga*. Modern systematics divide conifers I into Abietoideae (where *Abies* is) and Pinoideae, consisting of Lariceae (*Larix* + *Pseudotsuga*) and Pineae (*Pinus* + *Picea*) [6,46].

On the other hand, the structure of subclusters is significantly more complex compared to conifers II. The genomes of conifers I contain a much larger number of TEs, which leads to the formation of a large number of overlapping groups of elements. As a result, it becomes difficult to draw clear boundaries between individual subtrees.

This pattern likely reflects the superimposition of several waves of TE activity, leading to the formation of partially overlapping element lines. In this context, individual groups may correspond to lines that existed in common ancestors but were subsequently lost in some taxa. In some cases, such groups cannot be interpreted unambiguously.

A distinctive feature is the systematic appearance of independent groups of elements derived exclusively from a single genome, which cannot be grouped into subtrees due to the absence of elements from other genomes. Such cases occur in various parts of the tree, most frequently involving elements from *Abies*. It seems to be an extreme case of an individual TE lineage, with a following elimination in most taxa. These recurring groups can be regarded as separators because adjacent regions do not always contain a complete set of conifer representatives, which makes it difficult to interpret them as independent evolutionary units. Here we consider these separators as solo-survivor lineages, as mentioned in Figure 5C,D.

We selected 12 subtrees, without counting *Abies* + *Pseudotsuga* from *E-1* (two of five species were not informative) and the basal lineage from *E-4* that was too mixed. All other lineages highlighted in red in Figure 7 were considered as additional lineages in partially inappropriate positions. Lineages highlighted in blue were taken as parts of both surrounding subtrees. The main incongruence of all the selected subtrees of the conifer I group is that *Pinus* elements do not lie with *Picea* (as a part of *Pineae*) like *Larix* and *Pseudotsuga*, but are located either between *Abies* and *Picea* or closer to *Abies* than to *Picea*. Here, the hypothesis for the tree (*Abies*, (*Pinus*, *Picea*), (*Larix*, *Pseudotsuga*)) was not supported (0 cases of five full subtrees); however, the tree (*Abies*, (*Pinus*, *Picea*), (*Larix*, *Pseudotsuga*)) was more likely (five of five). Because of the discordance in *Pinus* TEs, we tested all 12 subtrees for the most distant separation of *Abies* from *Larix* and *Pseudotsuga*. The conditions that satisfied both trees were three cases where at least one of the three species wasn’t on a subtree, which were counted as negative (according to their uninformative nature). This condition was confirmed binomially (nine of 12, *p*-value = 4.75 × 10^−9^).

It is important to note that the observed complexity cannot be explained by either horizontal transfer or recombination events: the relevant elements generally do not show systematic mixing across species and retain their taxon-specificity. This indicates that vertical evolution, coupled with a high copy number and a long history of TE divergence, remains the primary factor shaping the observed topology.

### 3.5. Seed Plants Clusters: Tat E as a Main Copia-like Structure Representative Shows the Evolution of Gymnosperms Dividing the Conifer Groups

**Figure 8:**
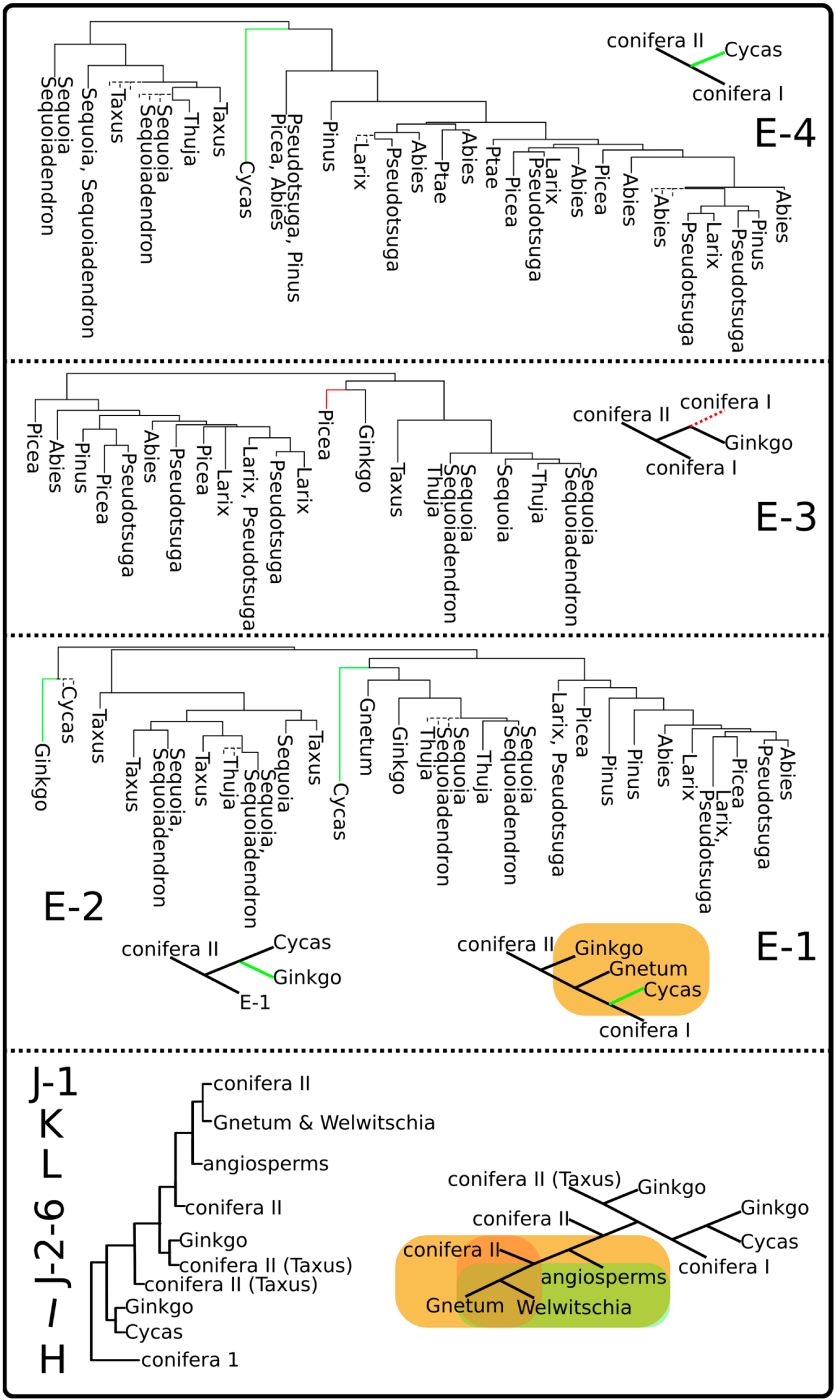
The main seed plant subclusters from the full *Tat* phylogeny (Figure 4) and their unrooted simplified subtrees. The scheme presents four subclusters of *Tat E* and clusters *H-L* as a giant subtree. All of the conifers I and conifers II clades are united. The green lines are highlighted in case of clades of small size (which could be counted both as artifacts and as remnants of oldest lineages). The ocher font highlights the Gnetophyta elements and their surrounding clades. In the case of the *E-1* subcluster, *Gnetum* is placed together with other ancient gymnosperms (*Ginkgo* and *Cycas*), while in the case of the *H-L* clusters, where Gnetophyta (both *Gnetum* and *Welwitschia*) appear only once, it lies closer to conifers II and angiosperms, meaning that some parts of the genome could also evolve like in the gnecup (orange font) and anthophyte (green font) scenarios. Together with the *E-1* subcluster case, Gnetophyta’s evolution seems mosaic, and the loss of *Welwitschia* elements in *Tat E* could also mean the loss of TE-neighboring genome loci. *Cycas* and *Ginkgo* are systematically placed closer, sometimes clustered together, sometimes independently, also showing mosaic patterns (between “Ginkgo + Cycas” and alternative hypotheses), where in some cases two groups could be considered as sister ones, or as separate taxa in another case. However, the additional clades of *Ginkgo* without *Cycas* in *J-5* could indirectly point out that *Ginkgo* is evolutionarily closer to conifers II, angiosperms, and Gnetophytes. The *J-5* and *J-6* subclusters (*Taxus* + *Ginkgo* and only *Taxus*, respectively) also reflect *Taxus*’ basal taxonomical place in conifers II, which has previously even been considered as a basal group to all gymnosperms.

The *Tat E* cluster consists of elements with a copia-like structure and is present in all seed plants except angiosperms. This distribution suggests that angiosperms occupy a distinct position within the diversity of *Tat* elements with this structure. However, the absence of corresponding elements for individual taxa does not necessarily indicate basal position or evolutionary remoteness of that taxon. Thus, as an example, in the second representative of the Gnetophyta—*Welwitschia*, whose genome has undergone a series of reduction events—elements of the *Tat E* cluster are not detected at all (they are detected only in a single *Tat K* cluster), whereas TEs from *Gnetum* are preserved. In this regard, the position of the Gnetophyta in this cluster is interpreted primarily based on *Gnetum* elements, suggesting that some TE lineages may have been secondarily lost in *Welwitschia*. Since there is no analogous representative for angiosperms, we examine cluster *Tat E* in terms of the relationships among the remaining seed plants (Figure 8).

*Tat E* also identifies several basally located clades from the *Ginkgo* and *Abies* genomes, the interpretation of which was discussed previously. These could be counted as separators and may presumably reflect remnants of more ancient lineages of elements that existed in the common ancestors of the respective groups. In addition to these, four main subclusters are identified, differing in their taxonomic composition.

Subcluster *E-1* consists of the elements conifer I, conifer II, *Ginkgo*, *Gnetum*, and a small *Cycas* lineage, which, due to its small size, can be considered either as an artifact or as a remnant of the oldest lineage. The latter has low node support (82/81), which is still reliable by the aLTR criterion, not mixed with other species, and located at the place it is supposed to be, making it no longer an artifact. After the transformation into an unrooted reconstruction, the two groups of conifers occupy extreme positions, with ancient gymnosperms located between them (Figure 8). The position of the small *Cycas* lineage remains uncertain: it can be regarded as an artifact or as a remnant of a more ancient lineage of taxa. However, the low support for the corresponding node does not allow for a confident interpretation of its position. As a result, the relationships between *Cycas*, *Ginkgo*, and *Gnetum* within this subcluster remain ambiguous, and their branching order should be treated with caution.

Subcluster *E-2* consists solely of conifers II elements, as well as basal *Ginkgo* and *Cycas* elements grouped together. *Ginkgo* is presented only by one lineage, has only 60% of its RT domain length, and has non-reliable node support (69/83), uniting it with *Cycas*. All these characteristics are not as sufficient as for *Cycas* from *E-1*, but are enough to not to be counted clearly as an artifact. At the same time, within the cypresses, the expected distribution corresponding to the scheme ((*Sequoia* + *Sequoiadendron*) + *Thuja*) + *Taxus*) is reproduced, which further indicates the preservation of the phylogenetic signal at the TE level. Subcluster *E-2* is virtually identical to *E-1* (Figure 8), except that it lacks the conifer I elements. It can also be considered together with *E-1*, by representing them as two levels of taxon-specific branching, where, in the basal case, conifer I either did not evolve or was lost.

Subcluster *E-3* consists of two groups of conifers, with the *Ginkgo* clade situated between them, once again highlighting the distance between conifer I and conifer II. A similar pattern is observed in subcluster *E-4*, where a small *Cycas* lineage is located between the two groups of conifers. Despite the small size of this lineage and low node support (66/50 compared to both relative conifer groups, >95/95, and the common node that united three of them, 94/93), its position is consistent with the distribution observed in the previous subcluster and may reflect remnants of an older state of the whole subcluster.

Taken together, these subclusters demonstrate that elements with a copia-like structure maintain a stable division between the two groups of conifers, with ancient gymnosperms occupying an intermediate position between them. This pattern may reflect the preservation, across different TE lineages, of signals corresponding to the early stages of seed plant divergence.

### 3.6. Seed Plants Clusters: Retroviral and Post-Integrase Structures of Tat H-L Unite Gnetophyta with Conifers II and Angiosperms

The second major branch of seed plants (*Tat H-L*) includes elements with retroviral and post-integrase structures, as well as a small group of *Taxus* elements with a copia-like structure situated between them. Two clusters with post-integrase structures occupy basal positions in this clade: one is represented by conifer I elements (*Tat H*) and is located closest to the *Tat* E clade, while the second includes *Ginkgo* and *Cycas* elements (*Tat I*).

Between the retroviral and post-integrase structures lies a distinct subcluster of *Taxus* elements with a copia-like structure (*Tat J-6*). Its position may correspond to an intermediate state between the two main branches, and potentially reflects a region of ancient recombination events associated with the formation of various *Tat* structures in this part of the tree. However, due to the uniqueness of this structure in *Taxus*, its interpretation remains preliminary.

Following this cluster, a series of subclusters with a retroviral structure is formed, including:

(a) The *Ginkgo* and *Taxus* subclusters (*Tat J-5,6*);
(b) The Gnetophyta cluster (*Gnetum* and *Welwitschia*, *Tat K*);
(c) The conifer II subclusters (*Tat J-1-4*);
(d) The angiosperm cluster (*Tat L*).

The first thing that stands out is the formation of a general clade comprising the Gnetophyta (represented by both species studied), conifers II, and angiosperms, which are represented once within the entire seed plant clade. Such a topology allows for multiple interpretations. On the one hand, the proximity of Gnetophyta to conifers II is consistent with the gnecup hypothesis. On the other hand, the inclusion of angiosperms in this group does not allow for the complete exclusion of scenarios similar to the anthophyte hypothesis. Here we should consider another interpretation about this “triumvirate”: absent taxa could have previously taken place in there but were evolutionarily lost. It still means some kind of evolutionary trends for Gnetophyta and angiosperms to evolve from basal gymnosperms (together with *Ginkgo* and *Cycas*) closer to conifers II have occurred.

At the same time, conifers II are represented by several additional lineages situated between the *Taxus* copia-like subcluster (*J-6*) and the group comprising gymnosperms and angiosperms. This may reflect the preservation of pre-existing intermediate TE lines corresponding to different stages of divergence of ancient seed plants.

The position of the *Taxus* elements also warrants separate consideration. In addition to its general relationship with conifers II, *Taxus* is represented by an additional subcluster with a copia-like structure, located between branches with retroviral and post-integrase structures. Some studies have suggested that Taxaceae represent a basal lineage relative to the remaining conifers [13], with cypresses and pines grouped together. The presence of a separate *Taxus* subcluster with the copia-like structure (similar to the closest basal cluster *Tat E*) is, therefore, consistent with our interpretation that this lineage occupies the position closest to the common ancestor of the entire *Tat H-L* branch.

The position of *Ginkgo* and *Cycas* deserves special attention. In the most basal parts of the branch, they are represented by elements with a post-integrase structure, which aligns them with conifers I, whereas further along a separate *Ginkgo* subcluster with a retroviral structure appears, located closer to gnetophytes, angiosperms, and conifers II. The absence of *Cycas* elements in this subcluster can be explained either by the secondary loss of the corresponding lineages or by differences in the degree of phylogenetic proximity with *Ginkgo*. Elements of both *Ginkgo* and *Cycas* lay closer to the theoretical root of the *Tat H-I* and *Tat J-K* clusters, supporting their place as ancient gymnosperms.

Overall, this clade may reflect a separation between two groups of conifers by ancient gymnosperms, while simultaneously forming a clade comprising gnetophytes (*Tat K*) and angiosperms (*Tat L*) clusters together with a *Tat J-1* subcluster of conifers II. The resulting picture does not allow for unequivocal support of any of the existing hypotheses regarding the origin of seed plants, but underlines the place of ancient gymnosperms separating two groups of conifers, while there are some signs of Gnetophyta and angiosperms getting closer to conifers II. Altogether, this points to the presence of a complex and partially mosaic signal preserved in the evolution of *Tat* elements.

### 3.7. The Tat Branch of Seed Plants: The General Verdict

A comprehensive analysis of *Tat* clades in seed plants reveals a robust, albeit heterogeneous, phylogenetic signal. Despite differences among individual subclusters and structures, most reconstructions reproduce a number of recurring patterns.

First and foremost, *Ginkgo* and *Cycas* are most often grouped together, which is consistent with the “Ginkgo + Cycas” hypothesis and does not support scenarios of their complete divergence into independent ancient lineages. At the same time, a partial divergence in their positions is observed in a number of subclusters: *Cycas* elements tend to group closer to conifers I, whereas *Ginkgo* elements are placed or have additional lineages closer to conifers II, gnetophytes, and angiosperms. Such a distribution may reflect both differences in the preservation of individual TE lineages and the heterogeneity of the phylogenetic signal itself.

Within the conifer II clade, *Taxus* TEs occupy the most isolated position. In a number of *Taxus* clades, elements are either positioned basally relative to the other members of the group or form independent branches separated from the cypresses. This distribution is consistent with the view that the Taxaceae diverged earlier within the Pinophyta that may reflect the preservation of ancestral states in individual *Tat* lineages.

Contrary to the expected close proximity of the two groups of conifers, in many subclusters, *Ginkgo* and *Cycas* are sequentially positioned between conifers I and conifers II. Furthermore, conifers II demonstrate an additional connection with the group comprising gnetophytes and angiosperms, placing them in a common set of clades with a retroviral structure. This topology allows for interpretations that are partially consistent with both the gnecup hypothesis and the anthophyte hypothesis about the position of gnetophytes. The option of the early presence of conifers I in this connection is still possible but assumes the fact of their elimination and coevolution of preserved groups.

Thus, *Tat* elements exhibit a complex and partially mosaic distribution of phylogenetic patterns. The different subclusters reproduce recurring patterns of taxon relationships that partially correspond to various existing hypotheses of plant systematics. Such a distribution may reflect the heterogeneity of the evolution of individual TE lineages and their associated genomic loci during the early stages of seed plant divergence. In this context, *Tat* elements can be viewed as an additional source of information on the relationships among major plant taxa, allowing for the analysis of alternative scenarios of their early evolution.

### 3.8. Non-Seed Plant Branch and Lycophytes Clusters Basal to Seed Plant Branch Suggest Another Interpretation of Bryophytes’ Evolution

First, it should be noted that the copy number of TEs from non-seed plants is less numerous than from seed plant genomes. This should be explained by a set of whole-genome duplication events [47,48,49] and the tendency of genome size accumulation in higher plant taxa evolution [50,51,52]. That is why only one subtree can be presented from non-seed plant *Tat* clusters.

**Figure 9:**
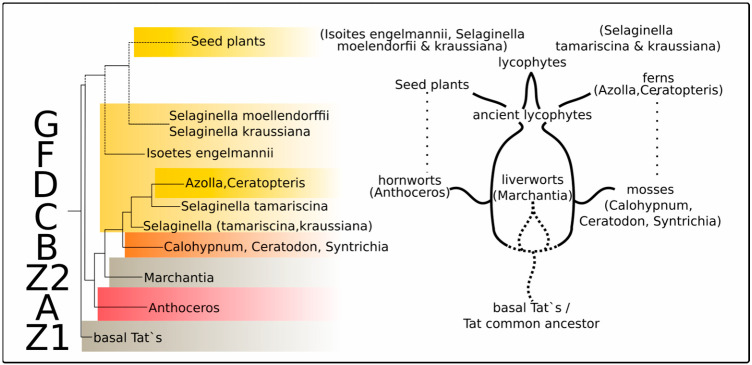
A schematic representation of the non-seed plant *Tat* TE reconstruction (**left**), with the following unrooted tree of the main plant groups, according to the evolution of *Tat* LTR retrotransposons (**right**). A common *Tat* ancestor (remaining as the *Tat* Z1 basal branch with a “zero” structure) existed at the stage of the common ancestor of “bryophytes”. Liverworts originated first, before aRNH acquisition. Then the “pool” of the *Tat* genetic and structural variants (saved in modern lycophytes) evolved differently by structure fixation or elimination, followed by branching of hornworts and mosses at first, ferns and seed plant ancestors later, and the mutually exclusive loss of *Tat* lineages. Here, hornworts are closer to seed plants, while mosses can be considered as closer non-lycophyte ancestors of ferns.

If we exclude the *Tat* clusters of lycophytes (*Tat F* and *G*), which are located basal to the seed plant clusters (*Tat E* and *H-L*), the distribution of *Tat* elements among non-seed plants appears quite consistent. Liverworts, hornworts, and mosses, as well as ferns, form distinct clusters with structure-specificity of almost every group. Meanwhile, paraphyletic lineages of lycophytes, represented by elements with various *Tat* structures, are situated between mosses and ferns (Figure 9, left).

This distribution generally aligns with a scenario of long-term vertical evolution of *Tat* elements following the divergence of the major groups of terrestrial plants. Except for the lycophytes, each group is represented primarily by a single large cluster and a single structure, indicating the long-term independent evolution of the corresponding TE lineages.

Of particular interest are the liverworts, whose elements retain a “zero” structure without an additional RNH domain (*Tat Z2*). Since a similar structure state is characteristic of the *Athila* group—a sister group to *Tat*—for the previously found basal *Tat* branch with no aRNH (*Z1*) and most of Ty3/Gypsy LTR retrotransposons, this structure can be considered as the ancestral state of *Tat* before the acquisition of aRNH. In this respect, the position of the liverworts corresponds to one of the most ancient preserved evolutionary lineages of *Tat* in terrestrial plants. That is why we consider the liverworts as the first and earliest taxa to offshoot during the aRNH acquisition events, followed by recombinations that gave rise to the discovered structures and their fixation in various taxa. In the case of hypotheses where it is proposed as sister to one of the other bryophyte groups, they should contain elements with the same structure nearby. Thus, we have developed a little argument to reject such a scenario with our data.

If we present this non-seed plant block as a simplified tree (Figure 9, right), then the remaining groups of plants diverge from the liverworts cluster (*Tat Z2*) in two directions. In one direction are the hornworts (*Tat A*), followed by two lycophyte clusters (*Tat F* and *G*) that are basal to the seed plant clusters (*Tat E* and *H-L*). In the other direction are mosses, paraphyletic lines of lycophytes (*Tat C*), and their sister group, the ferns (*Tat D*). This distribution is particularly interesting because the lycophyte clusters are linked to two distinct branches of *Tat* evolution in plants.

At the same time, lycophytes are the only group studied that exhibit all major variants of *Tat* structures containing aRNH. Furthermore, different *Tat* lineages in lycophytes occupy distant positions within the tree and are linked to various groups of plants. Some of them (*Tat C*) are situated between mosses (*Tat B*) and ferns (*Tat D*), while two other clusters are located further than hornworts (*Tat A*) and occupy a basal position relative to the large branch of seed plants (*Tat E*, *H-L*), including the most ancient of the known copia-like structures.

This paraphyly of lycophyte *Tat C* and *F-G* clusters allows for several interpretations. The simplest explanation would be multiple events of horizontal transfer. However, as within most other *Tat* clusters, there are no convincing signs of recent horizontal transfer here: the lineages retain their own structural specificity, do not show widespread mixing between taxa, and are often represented by clusters with high copy numbers that have evolved within their respective genomes.

In this regard, a more plausible scenario is one in which ancient lycophytes simultaneously preserved several evolutionarily distinct *Tat* lineages associated with different traces of early vascular plant evolution. In this case, modern lycophytes can be viewed as a group that has preserved fragments of ancient *Tat* diversity, which was subsequently lost in different ways among other taxa.

It is precisely in this context that the distribution of the two main branches of the *Tat* lineage is particularly interesting. On the one hand, a sequence of “mosses → lycophytes → ferns” (or *B → C → D*) can be traced, and on the other, “hornworts → lycophytes → seed plants” (*A → F + G → E + H-L*). This pattern does not necessarily reflect the direct phylogeny of the taxa themselves, but it may indicate that different *Tat* lineages were associated with different evolutionary trajectories within early vascular plants and their predecessors.

Under this interpretation, ancient lycophytes may have contained several large sets of *Tat* lineages that coexisted within shared genomes and were subsequently preserved in different ways among their descendants. Collectively, they (and their associated genome regions) formed a kind of “pool” of genetic diversity in the common ancestor of hornworts and mosses, as well as lycophytes and their later derived taxa. One part of the *Tat* lineages from the “pool”, associated with elements closely related to modern mosses and ferns, may have been gradually lost on the path to seed plants, whereas the other, conversely, may have been preserved and formed the main diversity of *Tat* in seed plants. For ferns, the opposite scenario may have played out.

We admit that such an interpretation remains largely hypothetical. However, it allows us to explain several observed features simultaneously: the paraphyly of lycophyte *Tat* lineages, the presence of all major *Tat* structures in them, and their intermediate position among different groups of plants.

## 4. Materials and Methods

### 4.1. Genome Assemblies

Genome assemblies at the chromosome or chromosome-like scaffold level were taken as described in a previous study [39] from a list of databases. Some genomes were excluded to avoid tree overloading. A shortened list of species is presented in Table S1.

### 4.2. Extraction of Tat LTR Retrotransposon Sequences

We used our previously written algorithm DARTS and its paradigm [53] as a pipeline to search TE copies with the following extraction of RT domains for subsequent clustering via MMseqs2 (“easy-cluster” command, 80% of identity) [54] and phylogenetic reconstruction. The main points are: (1) the search for the “central” (or so-called “core”) LTR retrotransposon domain (reverse transcriptase, RT); (2) the extraction of the domain with flanking regions; (3) the second round of searching for other domains common for TEs of the current group; and (4) the extraction of sequences and the order of domains for subsequent (5) clusterization, (6) annotation, and (7) phylogenetic reconstruction. The DARTS [53] sequence annotations are named (in File S1) as follows: “*%num_%g_%ID_%str_%score*”, where num—number of elements, clustered with with representative (clusterization from 3 elements to tens of thousands of Tat TE copies that are >80% identical to the RT domain helps to not overload the tree); g—genome short name; str—domain order structure; and ID and score—DARTS’ inner ID and score.

### 4.3. Phylogenetic Reconstruction

The basis for the RT tree phylogeny was taken from GyDB [55] of the Gypsy LTR-RTs. The multiple alignment of the extracted RT sequences was performed as a basis using MAFFT v. 7.505 [56]. The phylogenetic tree was built in IQ-Tree [57] with “-m TEST” (autodefinition of evolutionary model) and nodes supported by approximate likelihood-ratio test (aLRT) and ultrafast bootstrap (ufBooT) with 1000 iterations. For visualization and to import into PDF and SVG, the Figtree software v1.4.4 (https://github.com/rambaut/figtree/, accessed on 10 February 2025) was used. The output phylogenetic tree is presented in the Supplementary Material as File S2, together with the IQ-Tree log (File S3).

Because of too-close TE lineages inside clusters and subclusters, we used at least one of two 80/95 coefficients for node support at the clusters and subclusters levels, while more lateral levels were separated to species-specific and mixed clades by either 80/95 node support or by the existence of a “core” (lineages with high number of representatives after clusterization) together with a “shell” (at least a few lineages with small number of representatives in one branch with a “core” from same species).

### 4.4. Subtree Selection Steps

At the start, we split our phylogenetic tree from the *Tat* RT domain into previously separated [39] clusters (according to the huge taxa + structure relations).

After that, we went from the lateral leaves of the tree, searching for species-specific clades that are not mixed with other ones. We called clades mixed if we were unable to separate them either by node support values or by visual traits like strong differentiation in the group of lineages by the same species or by branch length. If there were one or two artifact lineages (all criteria listed in the “conceptualization” block) from different species inside the species-specific clade, they were ignored. In the case of higher quality or number of copies (i.e., a formed “core” and “shell”), such TEs were counted as a part of the clade that is now reclassified as mixed. A species-specific clade was counted as high-quality when at least one lineage of more than 100 copies (“core”) and no less than two lineages of less than 20 copies lay around (“shell”) were present, with at least one of two 80/95 node support values. However, clades with obvious edges, based on the abovementioned visual traits, and the presence of a “core + shell” were counted even without node support, reflecting close relations between clades.

The subtrees were selected after being divided into mixed and species-specific small clades. The basic rules that we use were:

(1) Clades should be grouped into subtrees by close relative species. That means that if previously there were only clades from conifers I, and then a clade of *Ginkgo* or *Cycas* (considered as another group of taxa) is added as basal, we should first check clades before it for the possibility of nested subtrees.
(2) We perform a search for a set of species-specific clades for the examined group. For an example of conifers I, it is expected to find a clade for each species (or some of them) and mixed clades around specific clades of their taxa (as described in Figure 5A). If mixed clades correspond to scenarios of their possible location, we exclude them from analysis but count them as verification of the close relation among their species-specific clades. Each subtree should, theoretically, consist of no more than 1 species-specific clade for each species. However, sometimes there are a number of clades from one species that lie together without other species between them. As part of the normal scenario for TE evolution (discussed in Figure 5 and the “species-specific tendency at genus level represents host’s relations” block), we count them either as one clade or as separators if clades separate two sets of species-specific clades with almost the same taxa representatives.
(3) If a set of clades we found before a new greater taxa added is single (no nested sets), we expand the set by new taxa until some species repeat. The recurrence of any species for the second time in different parts of the future subtree is the main reason for trees to be split.
(4) In cases of nested trees or repeated clades of the same species in different locations, this leads us to separate them into final subtrees. Here we use the taxa sets, dividing them by (a) separator clades, (b) differentiation by strong node support values, and (c) the length of the branches.

### 4.5. Quantitative Assessment of Host Topology Recovery

To quantitatively assess whether *Tat* subtrees reproduce the accepted host phylogeny more frequently than expected by chance, we performed a binomial enrichment test. For each independently interpreted *Tat* subtree within conifer I and conifer II, we recorded whether its topology matched the accepted host topology. Under the null hypothesis, all possible unrooted topologies were assumed to be equally likely (p_0_ = 1/3 for four-taxon subclusters and p_0_ = 1/15 for five-taxon subtrees). The observed number of matching subtrees was evaluated using a one-sided exact binomial test.

Because subtrees originate from the same TE family, they cannot be considered fully independent observations. Therefore, the binomial test is intended as an enrichment analysis providing quantitative support for the observed pattern rather than as a strict test of statistical independence.

## 5. Conclusions

### So, Would It Be Possible to Use TEs as Phylogeny Markers?

Overall, the results indicate that, under predominantly vertical inheritance, *Tat* elements are capable of retaining a distinct phylogenetic signal over long evolutionary time scales. Despite the mobile nature of these elements, most large clusters demonstrate stable structural and taxonomic specificity, and their distribution in many cases is consistent with known host relationships.

In this context, *Tat* lineages can be viewed not only as transposable elements, but also as a collection of numerous independent evolutionary markers, some of which have co-evolved with various host genome regions, whose evolution arouses interest to be studied additionally. At the same time, different TE lineages may retain traces of different stages of divergence and respond differently to processes of selection, recombination, and loss. As a result, individual reconstructions may partially correspond to different existing hypotheses on plant systematics, reflecting the complexity and mosaic nature of the evolutionary processes underlying the early divergence of major plant taxa.

One important conclusion of this work is methodological rather than phylogenetic. The proposed interpretation of transposable element gene trees as collections of partially independent lineage histories appears capable of recovering known host relationships in well-sampled groups. Future studies with denser taxon sampling and automated subtree recognition will be required to establish the statistical properties and broader applicability of this approach.

Of course, this approach requires careful interpretation. Horizontal transfer, recombination events, differences in the rates of lineage degradation, and the incomplete preservation of ancient elements can significantly distort the observed picture. Nevertheless, in cases where it is possible to trace the predominantly vertical evolution of highly duplicated TE lineages, such elements can serve as an additional source of information for reconstructing complex evolutionary events that are difficult to resolve using a limited number of classical molecular markers.

Nevertheless, for most of the analyzed plant groups, the clusters exhibit obvious species- and taxon-specificity: the distribution of elements in conifers (of both groups) generally agrees with the phylogeny of their hosts. This makes it unlikely that horizontal transfer has made a significant contribution at the family and genus levels, as well as for the majority of non-seed plants. At the same time, its possible contribution, along with recombination events, cannot be completely ruled out at deeper stages of divergent evolution—in particular, during the divergence of seed plants and in the evolution of lycophytes. However, these findings are enough to support host phylogeny interpretations that comprise some confronting hypotheses on current plant group evolution.

## Data Availability

The original contributions presented in this study are included in the Supplementary Materials. Further inquiries regarding intermediate data can be directed to the corresponding author.

## Supplementary Materials

The following supporting information can be downloaded at: https://www.mdpi.com/article/10.3390/plants15152388/s1. Table S1: List of selected genomes, their taxonomy, assembly, and found lineages information; File S1: Multiple alignment of RT domains; File S2: Phylogenetic tree file; File S3: IQ-Tree log file; Figure S1: Full version of Figure 4 corresponding to File S2; and Figure S2: The idea of the “core-and-shell” of clades in part of the J-1 subcluster example.

## Abbreviations

The following abbreviations are used in this manuscript:

TEs	Transposable Elements
RNH	Ribonuclease H
aRNH	Additional Ribonuclease H
HT	Horizontal transfer

## References

[1] Harris B.J., Clark J.W., Schrempf D., Szöllősi G.J., Donoghue P.C.J., Hetherington A.M., Williams T.A. (2022). Divergent Evolutionary Trajectories of Bryophytes and Tracheophytes from a Complex Common Ancestor of Land Plants. Nat. Ecol. Evol..

[2] Laenen B., Shaw B., Schneider H., Goffinet B., Paradis E., Désamoré A., Heinrichs J., Villarreal J.C., Gradstein S.R., McDaniel S.F. (2014). Extant Diversity of Bryophytes Emerged from Successive Post-Mesozoic Diversification Bursts. Nat. Commun..

[3] Morris J.L., Puttick M.N., Clark J.W., Edwards D., Kenrick P., Pressel S., Wellman C.H., Yang Z., Schneider H., Donoghue P.C.J. (2018). The Timescale of Early Land Plant Evolution. Proc. Natl. Acad. Sci. USA.

[4] Qiu Y.-L., Li L., Wang B., Chen Z., Knoop V., Groth-Malonek M., Dombrovska O., Lee J., Kent L., Rest J. (2006). The Deepest Divergences in Land Plants Inferred from Phylogenomic Evidence. Proc. Natl. Acad. Sci. USA.

[5] Qiu Y.-L., Mishler B.D. (2024). Relationships among the Bryophytes and Vascular Plants: A Case Study in Deep-Time Reconstruction. Diversity.

[6] Yang Y., Ferguson D.K., Liu B., Mao K.-S., Gao L.-M., Zhang S.-Z., Wan T., Rushforth K., Zhang Z.-X. (2022). Recent Advances on Phylogenomics of Gymnosperms and a New Classification. Plant Divers..

[7] Coiro M., Roberts E.A., Hofmann C.-C., Seyfullah L.J. (2022). Cutting the Long Branches: Consilience as a Path to Unearth the Evolutionary History of Gnetales. Front. Ecol. Evol..

[8] Wang X.-Q., Ran J.-H. (2014). Evolution and Biogeography of Gymnosperms. Mol. Phylogenet. Evol..

[9] Stull G.W., Qu X.-J., Parins-Fukuchi C., Yang Y.-Y., Yang J.-B., Yang Z.-Y., Hu Y., Ma H., Soltis P.S., Soltis D.E. (2021). Gene Duplications and Phylogenomic Conflict Underlie Major Pulses of Phenotypic Evolution in Gymnosperms. Nat. Plants.

[10] Liu Y., Wang S., Li L., Yang T., Dong S., Wei T., Wu S., Liu Y., Gong Y., Feng X. (2022). The Cycas Genome and the Early Evolution of Seed Plants. Nat. Plants.

[11] Ran J.-H., Shen T.-T., Wang M.-M., Wang X.-Q. (2018). Phylogenomics Resolves the Deep Phylogeny of Seed Plants and Indicates Partial Convergent or Homoplastic Evolution between Gnetales and Angiosperms. Proc. R. Soc. B Biol. Sci..

[12] Christenhusz M.J.M., Reveal J.L., Farjon A., Gardner M.F., Mill R.R., Chase M.W. (2011). A New Classification and Linear Sequence of Extant Gymnosperms. Phytotaxa.

[13] Yang Y., Yang Z., Ferguson D.K. (2024). The Systematics and Evolution of Gymnosperms with an Emphasis on a Few Problematic Taxa. Plants.

[14] Kapitonov V.V., Jurka J. (2001). Rolling-Circle Transposons in Eukaryotes. Proc. Natl. Acad. Sci. USA.

[15] Vitte C., Bennetzen J.L. (2006). Analysis of Retrotransposon Structural Diversity Uncovers Properties and Propensities in Angiosperm Genome Evolution. Proc. Natl. Acad. Sci. USA.

[16] Arkhipova I.R., Yushenova I.A. (2019). Giant Transposons in Eukaryotes: Is Bigger Better?. Genome Biol. Evol..

[17] Kim Y.-J., Lee J., Han K. (2012). Transposable Elements: No More “Junk DNA.”. Genom. Inform..

[18] Chénais B., Caruso A., Hiard S., Casse N. (2012). The Impact of Transposable Elements on Eukaryotic Genomes: From Genome Size Increase to Genetic Adaptation to Stressful Environments. Gene.

[19] Marino A., Debaecker G., Fiston-Lavier A.-S., Haudry A., Nabholz B. (2025). Effective Population Size Does Not Explain Long-Term Variation in Genome Size and Transposable Element Content in Animals. eLife.

[20] Schnable P.S., Ware D., Fulton R.S., Stein J.C., Wei F., Pasternak S., Liang C., Zhang J., Fulton L., Graves T.A. (2009). The B73 Maize Genome: Complexity, Diversity, and Dynamics. Science.

[21] Niu S., Li J., Bo W., Yang W., Zuccolo A., Giacomello S., Chen X., Han F., Yang J., Song Y. (2022). The Chinese Pine Genome and Methylome Unveil Key Features of Conifer Evolution. Cell.

[22] Kalendar R., Vicient C.M., Peleg O., Anamthawat-Jonsson K., Bolshoy A., Schulman A.H. (2004). Large Retrotransposon Derivatives: Abundant, Conserved but Nonautonomous Retroelements of Barley and Related Genomes. Genetics.

[23] Smýkal P., Kalendar R., Ford R., Macas J., Griga M. (2009). Evolutionary Conserved Lineage of Angela-Family Retrotransposons as a Genome-Wide Microsatellite Repeat Dispersal Agent. Heredity.

[24] Moisy C., Schulman A.H., Kalendar R., Buchmann J.P., Pelsy F. (2014). The Tvv1 Retrotransposon Family Is Conserved between Plant Genomes Separated by over 100 Million Years. Theor. Appl. Genet..

[25] Kalendar R., Tanskanen J., Chang W., Antonius K., Sela H., Peleg O., Schulman A.H. (2008). Cassandra Retrotransposons Carry Independently Transcribed 5S RNA. Proc. Natl. Acad. Sci. USA.

[26] Hassan A.H., Mokhtar M.M., El Allali A. (2024). Transposable Elements: Multifunctional Players in the Plant Genome. Front. Plant Sci..

[27] Hassan A.H., Mokhtar M.M., El Allali A., Garcia S., Nualart N. (2023). TEMM: A Curated Data Resource for Transposon Element-Based Molecular Markers in Plants. Plant Genomic and Cytogenetic Databases.

[28] Khedkar S., Smyshlyaev G., Letunic I., Maistrenko O.M., Coelho L.P., Orakov A., Forslund S.K., Hildebrand F., Luetge M., Schmidt T.S.B. (2022). Landscape of Mobile Genetic Elements and Their Antibiotic Resistance Cargo in Prokaryotic Genomes. Nucleic Acids Res..

[29] Roy N.S., Choi J.-Y., Lee S.-I., Kim N.-S. (2015). Marker Utility of Transposable Elements for Plant Genetics, Breeding, and Ecology: A Review. Genes Genom..

[30] Jing R., Vershinin A., Grzebyta J., Shaw P., Smýkal P., Marshall D., Ambrose M.J., Ellis T.N., Flavell A.J. (2010). The Genetic Diversity and Evolution of Field Pea (Pisum) Studied by High Throughput Retrotransposon Based Insertion Polymorphism (RBIP) Marker Analysis. BMC Evol. Biol..

[31] Kalendar R., Amenov A., Daniyarov A. (2018). Use of Retrotransposon-Derived Genetic Markers to Analyse Genomic Variability in Plants. Funct. Plant Biol..

[32] Deininger P.L., Roy-Engel A.M., Craig N.L., Craigie R., Gellert M., Lambowitz A.M. (2007). Mobile Elements in Animal and Plant Genomes. Mobile DNA II.

[33] Abrusán G., Krambeck H.-J. (2006). Competition May Determine the Diversity of Transposable Elements. Theor. Popul. Biol..

[34] Wallau G.L., Ortiz M.F., Loreto E.L.S. (2012). Horizontal Transposon Transfer in Eukarya: Detection, Bias, and Perspectives. Genome Biol. Evol..

[35] Wickell D.A., Li F.-W. (2020). On the Evolutionary Significance of Horizontal Gene Transfers in Plants. New Phytol..

[36] Wright D.A., Voytas D.F. (1998). Potential Retroviruses in Plants: Tat1 Is Related to a Group of Arabidopsis Thaliana Ty3/Gypsy Retrotransposons That Encode Envelope-like Proteins. Genetics.

[37] Ustyantsev K., Novikova O., Blinov A., Smyshlyaev G. (2015). Convergent Evolution of Ribonuclease H in LTR Retrotransposons and Retroviruses. Mol. Biol. Evol..

[38] Ustyantsev K., Blinov A., Smyshlyaev G. (2017). Convergence of Retrotransposons in Oomycetes and Plants. Mob. DNA.

[39] Biryukov M., Ustyantsev K. (2023). Origin and Evolution of Plant Long Terminal Repeat Retrotransposons with Additional Ribonuclease H. Genome Biol. Evol..

[40] Wang Q., Wang Y., Wang J., Gong Z., Han G.-Z. (2021). Plants Acquired a Major Retrotransposon Horizontally from Fungi during the Conquest of Land. New Phytol..

[41] Friesen N., Brandes A., Heslop-Harrison J.S. (2001). Diversity, Origin, and Distribution of Retrotransposons (Gypsy and Copia) in Conifers. Mol. Biol. Evol..

[42] Qiu F., Ungerer M.C. (2018). Genomic Abundance and Transcriptional Activity of Diverse Gypsy and Copia Long Terminal Repeat Retrotransposons in Three Wild Sunflower Species. BMC Plant Biol..

[43] Natali L., Cossu R.M., Mascagni F., Giordani T., Cavallini A. (2015). A Survey of Gypsy and Copia LTR-Retrotransposon Superfamilies and Lineages and Their Distinct Dynamics in the Populus Trichocarpa (L.) Genome. Tree Genet. Genomes.

[44] Soltis P.S., Soltis D.E. (2004). The Origin and Diversification of Angiosperms. Am. J. Bot..

[45] Leslie A.B., Beaulieu J., Holman G., Campbell C.S., Mei W., Raubeson L.R., Mathews S. (2018). An Overview of Extant Conifer Evolution from the Perspective of the Fossil Record. Am. J. Bot..

[46] Gernandt D.S., Vining T.F., Campbell C.S., Piñero D., Liston A. (1999). Molecular Phylogeny of Pinaceae and Pinus. Proceedings of the IV International Conifer Conference 615.

[47] Li Z., Baniaga A.E., Sessa E.B., Scascitelli M., Graham S.W., Rieseberg L.H., Barker M.S. (2015). Early Genome Duplications in Conifers and Other Seed Plants. Sci. Adv..

[48] Wan T., Liu Z.-M., Li L.-F., Leitch A.R., Leitch I.J., Lohaus R., Liu Z.-J., Xin H.-P., Gong Y.-B., Liu Y. (2018). A Genome for Gnetophytes and Early Evolution of Seed Plants. Nat. Plants.

[49] Jiao Y., Wickett N.J., Ayyampalayam S., Chanderbali A.S., Landherr L., Ralph P.E., Tomsho L.P., Hu Y., Liang H., Soltis P.S. (2011). Ancestral Polyploidy in Seed Plants and Angiosperms. Nature.

[50] He B., Liu W., Li J., Xiong S., Jia J., Lin Q., Liu H., Cui P. (2024). Evolution of Plant Genome Size and Composition. Genom. Proteom. Bioinform..

[51] Bennetzen J.L., Kellogg E.A. (1997). Do Plants Have a One-Way Ticket to Genomic Obesity?. Plant Cell.

[52] Michael T.P. (2014). Plant Genome Size Variation: Bloating and Purging DNA. Brief. Funct. Genom..

[53] Biryukov M., Ustyantsev K. (2022). DARTS: An Algorithm for Domain-Associated Retrotransposon Search in Genome Assemblies. Genes.

[54] Steinegger M., Söding J. (2017). MMseqs2 Enables Sensitive Protein Sequence Searching for the Analysis of Massive Data Sets. Nat. Biotechnol..

[55] Llorens C., Futami R., Covelli L., Domínguez-Escribá L., Viu J.M., Tamarit D., Aguilar-Rodríguez J., Vicente-Ripolles M., Fuster G., Bernet G.P. (2011). The Gypsy Database (GyDB) of Mobile Genetic Elements: Release 2.0. Nucleic Acids Res..

[56] Katoh K., Standley D.M. (2013). MAFFT Multiple Sequence Alignment Software Version 7: Improvements in Performance and Usability. Mol. Biol. Evol..

[57] Minh B.Q., Schmidt H.A., Chernomor O., Schrempf D., Woodhams M.D., von Haeseler A., Lanfear R. (2020). IQ-TREE 2: New Models and Efficient Methods for Phylogenetic Inference in the Genomic Era. Mol. Biol. Evol..

